# Editorial: Immune checkpoint inhibitors in renal cell carcinoma

**DOI:** 10.3389/fonc.2023.1203463

**Published:** 2023-04-18

**Authors:** Scott S. Tykodi, Renate Pichler

**Affiliations:** ^1^ Department of Medicine, Division of Medical Oncology, University of Washington and Clinical Research Division, Fred Hutchinson Cancer Center, Seattle, WA, United States; ^2^ Department of Urology, Comprehensive Cancer Center Innsbruck (CCCI), Medical University of Innsbruck, Innsbruck, Austria

**Keywords:** renal cell carcinoma, immune checkpoint inhibitor (ICI), immunotherapy, treatment free survival, biomarker, immune related adverse event (irAE)

Renal cell carcinoma (RCC) has long been viewed as a tumor with unique sensitivity to immunotherapies. The clinical development of systemic cytokines interferon-alpha or interleukin-2 as front-line therapy dating to the late 1980’s represented a unique treatment paradigm for metastatic carcinoma in a field dominated by use of cytotoxic chemotherapies. In the mid 2000’s, molecularly targeted therapies blocking vascular endothelial growth factor receptor or mammalian/mechanistic target of rapamycin signaling were rapidly embraced for front-line management of advanced disease for their higher response and disease control rates. However, the uniform development of resistant disease proved to be a significant limitation to this approach. The recent emergence of immune checkpoint inhibitors (ICIs) blocking PD1/PDL1 or CTLA4 signaling pathways has re-established systemic immunotherapy as central to the medical management of advanced RCC. A series of positive phase III trials of ICIs or ICI/tyrosine kinase inhibitor (TKI) doublet combinations, all showing clinical benefit including superior overall survival versus sunitinib monotherapy, have established the current treatment paradigm for advanced RCC (1). Thus, practitioners are now faced with selecting their preferred treatment from among four ICI-containing doublet regimens.

The widespread adoption of ICIs in cancer therapy has encouraged detailed analysis of their unique properties. In assessing efficacy, this drug class has been associated with early response heterogeneity with some patients showing a pattern of radiographic progression that precedes a subsequent response, often described as pseudoprogression (2). In addition, durability of tumor responses even after drug discontinuation, identified as treatment free survival (TFS), is an RCC phenotype more commonly associated with ICIs than with targeted therapies (3). However, despite the proportionally better success for ICIs or ICI/TKI combination regimens vs TKIs alone, outcomes remain imperfect with most patients ultimately experiencing treatment resistance and tumor progression, or drug intolerance. Biomarkers for ICI treatment efficacy are of great interest for RCC to guide optimal selection of available treatment options. ICIs have also been associated with a unique toxicity pattern versus other drug classes that reflects immune dysregulation and autoimmune pathology targeting normal tissues.

In the series of manuscripts responding to the Research Topic “Immune Checkpoint Inhibitors in Renal Cell Carcinoma”, the contributing authors address many key issues facing clinicians and researchers who treat RCC with ICIs and who study the immunobiology of this disease. Efficacy outcomes for advanced RCC treated by front-line ICI containing doublets are summarized by Tung and Sahu who provide a comprehensive review of the current therapy landscape and introduce ongoing clinical research investigating ICIs in combination with novel agents. Jo et al. present a single center retrospective study highlighting the association of International Metastatic renal cell carcinoma Database Consortium (IMDC) risk category with the proportional benefit of ICIs versus targeted therapy. They compare real world outcomes for IMDC poor risk RCC showing better efficacy endpoints with ICI-based combinations versus TKI monotherapies. Rebuzzi et al. utilizing data from the Meet-URO-15 multicenter retrospective study of nivolumab treated RCC patients, analyzed serial blood counts from clinical safety laboratory data to assess the associations of absolute cell counts and inflammatory ratios with clinical outcomes. On-treatment neutrophil increase and increasing neutrophil to lymphocyte ratio (NLR) were negatively associated with progression free survival (PFS) and overall survival (OS) representing dynamic prognostic factors with potential clinical utility for on-treatment decision making. Bimbatti et al. addressed TFS associated with ICIs reporting on a single institution cohort of 14 RCC patients who discontinued nivolumab in the absence of disease progression. Median PFS from the date of discontinuation was 19.8 months with treatment duration > 12 months and objective response associated with longer PFS. In addition, 3 patients were re-treated with nivolumab for disease progression, and all achieved subsequent disease stability.

Based on the current treatment paradigm for advanced RCC, most patients are treated with an ICI-based regimen in the front-line setting. The role and potential benefit for ICIs as salvage therapy for patient’s refractory to PD1 and/or CTLA4 blockade has not been well established in the context of prospectively enrolled, randomized, comparison clinical trials. Papathanassiou et al. conducted a systematic review compiling 10 studies totaling 500 RCC patients with ICI refractory disease who were treated with ICI-containing therapies in the second line or beyond. Aggregate efficacy outcomes showed an objective response rate (ORR) of 19% and PFS of 5.6 months with ≥ grade 3 adverse events seen in 25% of patients indicating modest efficacy and tolerable toxicity in this clinical context.

The discovery of predictive biomarkers for ICI mediated control of RCC has been an elusive target that encourages ongoing evaluation. Kim et al. report on the use of multiplexed immunohistochemistry to detect immune cell subsets in the tumor microenvironment from 24 RCC patients treated by nivolumab plus ipilimumab. Higher densities of Foxp3^-^CD4^+^ helper T cells, CD68^+^CD206^-^ M1 macrophages, and CD137^+^CD8^+^ cytotoxic T cells were associated with better PFS, with the Foxp3^-^CD4^+^ helper T cell association remaining significant in multivariate modeling. Yuan et al. report the development of a 13 gene signature for cuproptosis categorizing patients into high versus low-risk groups according to the median score. The low-risk phenotype was prognostic for better PFS and OS in The Cancer Genome Atlas data and associated with better PFS in patients treated with both ICI-based regimens and TKIs.

Immune related adverse events (IRAE) represent the unique toxicity profile of ICI-based treatment regimens. Many RCC patients have undergone nephrectomy surgery for management of the primary kidney tumor resulting in reduced renal functional capacity. Renal toxicities associated with ICIs are therefore of particular relevance to this population. Liu et al. address the incidence of renal adverse events (RAEs) for ICI-based regimens versus targeted or chemotherapies culled from 95 randomized controlled trials (including all cancer diagnoses) totaling > 40,000 patients. The overall incidence of ≥ grade 3 RAEs was 4.3%. Among ICI monotherapies, anti-CTLA4 had a higher risk of ≥ grade 3 renal adverse events (RAEs) than anti-PD1/PDL1. The anti-CTLA4/PD1 combination also had higher risk for RAEs than anti-PD1. Scarlotta et al. describe a case report of a patient treated for RCC with nivolumab plus ipilimumab who also had a history of diffuse large B cell lymphoma, in remission. The patient developed diffuse lymphadenopathy representing a diagnostic dilemma for this clinical presentation that could represent disease progression vs sarcoid-like autoimmunity versus infection. Diagnostic and management challenges for ICI associated toxicities are also addressed by Roberto et al. who highlight the value of a multidisciplinary approach to the management of high grade IRAEs.

## Author contributions

ST drafted the manuscript. Both authors edited, reviewed and approved the submitted version.
